# Artificial intelligence in the diagnosis of uveal melanoma: advances and applications

**DOI:** 10.3389/ebm.2025.10444

**Published:** 2025-02-19

**Authors:** Albert K. Dadzie, Sabrina P. Iddir, Sanjay Ganesh, Behrouz Ebrahimi, Mojtaba Rahimi, Mansour Abtahi, Taeyoon Son, Michael J. Heiferman, Xincheng Yao

**Affiliations:** ^1^ Department of Biomedical Engineering, University of Illinois Chicago, Chicago, IL, United States; ^2^ Department of Ophthalmology and Visual Sciences, University of Illinois Chicago, Chicago, IL, United States

**Keywords:** choroidal tumors, machine learning, deep learning, uveal melanoma, artificial intelligence, ocular oncology, convolutional neural networks

## Abstract

Advancements in machine learning and deep learning have the potential to revolutionize the diagnosis of melanocytic choroidal tumors, including uveal melanoma, a potentially life-threatening eye cancer. Traditional machine learning methods rely heavily on manually selected image features, which can limit diagnostic accuracy and lead to variability in results. In contrast, deep learning models, particularly convolutional neural networks (CNNs), are capable of automatically analyzing medical images, identifying complex patterns, and enhancing diagnostic precision. This review evaluates recent studies that apply machine learning and deep learning approaches to classify uveal melanoma using imaging modalities such as fundus photography, optical coherence tomography (OCT), and ultrasound. The review critically examines each study’s research design, methodology, and reported performance metrics, discussing strengths as well as limitations. While fundus photography is the predominant imaging modality being used in current research, integrating multiple imaging techniques, such as OCT and ultrasound, may enhance diagnostic accuracy by combining surface and structural information about the tumor. Key limitations across studies include small dataset sizes, limited external validation, and a reliance on single imaging modalities, all of which restrict model generalizability in clinical settings. Metrics such as accuracy, sensitivity, and area under the curve (AUC) indicate that deep learning models have the potential to outperform traditional methods, supporting their further development for integration into clinical workflows. Future research should aim to address current limitations by developing multimodal models that leverage larger, diverse datasets and rigorous validation, thereby paving the way for more comprehensive, reliable diagnostic tools in ocular oncology.

## Impact statement

Machine learning and deep learning are reshaping oncology diagnostics, yet applications in uveal melanoma remain underexplored, particularly in using multimodal imaging to improve accuracy. This review highlights significant gaps in current research, such as the over-reliance on single imaging modalities and limited datasets, which restrict diagnostic precision, generalizability, and clinical utility. By identifying these limitations and proposing multimodal integration as a viable solution, this work advances the understanding of how diverse imaging data can be effectively leveraged for ocular tumor detection. This new perspective provides a foundation for developing robust, cross-validated models that could transform diagnostic practices, enabling early and reliable identification of uveal melanoma. The insights presented here set a clear direction for future research to refine and implement comprehensive, automated diagnostic tools in ocular oncology, enhancing clinical decision-making and patient outcomes.

## Introduction

Melanocytic choroidal tumors encompass a spectrum of intraocular lesions that range from benign choroidal nevi to malignant melanomas. While choroidal nevi are common and typically asymptomatic, choroidal melanoma, though rare, are associated with a significant risk of metastasis and poor outcomes [1–5]. Early detection and accurate differentiation between these lesions are critical for management decisions and improving patient outcomes [6].

The diagnosis of melanocytic choroidal tumors is based on clinical examination and imaging techniques such as fundus photography, optical coherence tomography (OCT), and ultrasonography. Ophthalmologists assess features such as tumor size, thickness, and the presence of risk factors like subretinal fluid, orange pigment, and drusen to estimate the likelihood of malignancy [7, 8]. However, these diagnostic techniques are subject to inter-observer variability, and their sensitivity in detecting small melanomas, which can closely resemble benign nevi, is limited. In some cases, intraocular biopsies may be necessary to confirm the diagnosis, but these procedures carry some risks. This creates an unmet need for more objective, reproducible, and accurate diagnostic tools.

In recent years, artificial intelligence has emerged as a transformative technology in medical imaging. Machine learning, a subset of artificial intelligence, has shown great potential in automating diagnostic tasks that previously required expert interpretation. Deep learning, a further subset of machine learning, utilizes neural networks that can automatically learn and extract features from large datasets without the need for manual feature engineering. Convolutional neural networks (CNNs), in particular, have demonstrated impressive performance in image classification tasks across various medical fields, including ophthalmology [9–12].

This review aims to provide an overview of the current applications of artificial intelligence in the diagnosis of melanocytic choroidal tumors. It will discuss recent developments in artificial intelligence-based diagnostic models, the integration of multimodal imaging techniques, and the potential of these technologies to improve diagnostic accuracy and patient management. Furthermore, the review will address the limitations and challenges faced by machine learning applications in this field, as well as future directions for research and clinical translation.

## Overview of melanocytic choroidal tumors

Melanocytic choroidal tumors represent a spectrum of similar appearing lesions located in the choroid, a vascular layer beneath the retina. These tumors can be broadly classified into benign choroidal nevi and malignant melanoma. The accurate differentiation between these two forms is crucial, as their management and prognosis differ dramatically.

### Choroidal nevi

**FIGURE 1:**
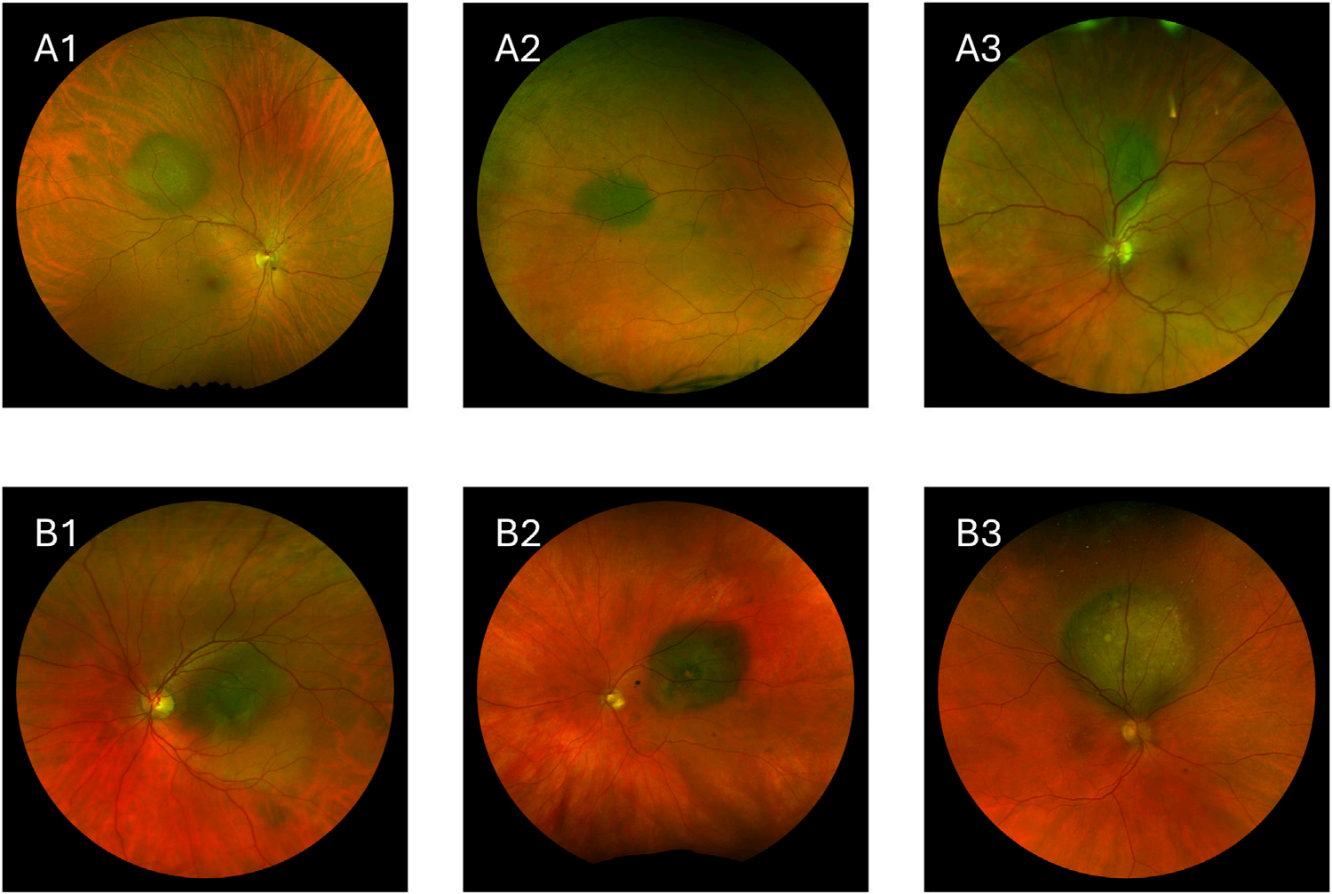
Images showing choroidal nevi and choroidal melanoma. **(A1–A3)** Choroidal nevi. **(B1–B3)** Choroidal melanoma.

Choroidal nevi (Figures 1A1–3) are benign lesions that are more commonly found in the White population compared to other ethnic groups [13–15]. These nevi are typically asymptomatic and remain stable over time, often being discovered incidentally during routine eye exams [16]. However, while most nevi remain benign, a small percentage can undergo malignant transformation into melanoma [17]. The risk factors that suggest potential malignancy include greater lesion thickness, subretinal fluid, orange pigment, and the absence of overlying drusen [17–19].

### Choroidal melanoma

Choroidal melanoma, the most common primary intraocular malignancy in adults, is a rare but aggressive disease with high metastatic potential (Figures 1B1–3) [20–22]. Approximately 50% of patients diagnosed with choroidal melanoma develop metastatic disease, with a high mortality rate once metastasis occurs [23, 24].

Choroidal melanomas are generally larger and thicker than nevi, and they often exhibit clinical features such as orange pigment, subretinal fluid, and low internal echogenicity. Advanced melanomas may also show a “mushroom” shape due to the tumor breaking through Bruch’s membrane [25]. Traditional diagnostic methods rely on clinical examinations, imaging modalities, and tools like the MOLES algorithm [2] to identify these features, but distinguishing between benign and malignant lesions, especially in cases with overlapping features of nevi and melanoma, can be challenging [26]. However, patients referred to specialist ocular oncology centers may benefit from higher diagnostic accuracy due to the expertise and advanced diagnostic resources available. Figure 1 shows how visually similar benign and malignant lesions can be.

### The need for improved diagnostic tools

One clinical challenge lies in identifying small choroidal melanomas that may closely resemble benign nevi in terms of clinical and imaging features. Misclassification can lead to either overtreatment or delayed treatment. Overtreatment exposes patients to unnecessary procedures such as radiation therapy, which carries risks of vision loss and other complications, while delayed treatment increases the risk of metastasis [27–29]. In some cases, intraocular biopsies may be necessary to confirm the diagnosis; however, these procedures carry risks such as hemorrhage, retinal detachment, and endophthalmitis [30, 31].

Given the clinical and imaging challenges, there is a critical need for more objective and precise diagnostic tools. This is where machine learning offers great promise. These advanced algorithms can process large datasets of imaging data to detect subtle features that may not be easily discernible by human experts, potentially revolutionizing the accuracy and efficiency of diagnosing melanocytic choroidal tumors.

## Overview of artificial intelligence

**FIGURE 2:**
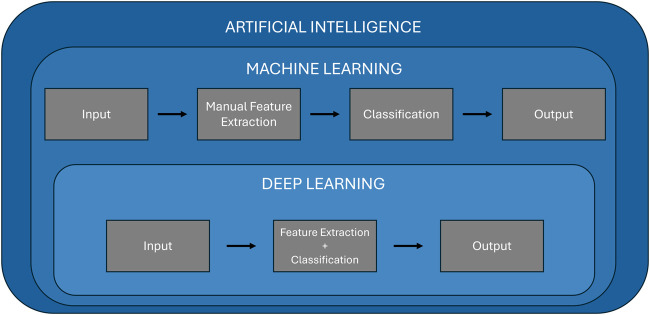
Relationship and differences between machine learning and deep learning.

Artificial intelligence refers to the ability of machines to mimic human intelligence, enabling them to perform tasks such as learning, problem-solving, and decision-making. Machine learning is a subset of artificial intelligence that enables computers to learn from data and make predictions or decisions without being explicitly programmed for every possible scenario. Machine learning algorithms build models based on patterns identified in training data, which can then be applied to new, unseen data for predictions. The key advantage of machine learning lies in its ability to adapt and improve over time as it processes more data, making it highly valuable for complex tasks like medical diagnosis. Over time, machine learning has evolved to include deep learning, a subfield of machine learning that leverages neural networks to model intricate relationships in data, particularly in fields like computer vision and natural language processing. Figure 2 illustrates the hierarchical relationship between artificial intelligence, machine learning, and deep learning. It highlights the key differences in processing workflows, showing that machine learning involves a manual feature extraction step, whereas deep learning integrates both feature extraction and classification within a single, automated framework.

### Machine learning

Machine learning models can generally be classified into four primary categories: supervised learning, unsupervised learning, semi-supervised, and reinforcement learning. Supervised learning, the most common type, involves training models on labeled datasets, where each data point is associated with the correct output. Algorithms like support vector machines (SVMs), random forest, and K-nearest neighbors (KNNs) are widely used in supervised learning to perform tasks such as classification and regression. In contrast, unsupervised learning deals with unlabeled data, and the goal is to uncover hidden patterns within the data. Techniques like K-means clustering, and principal component analysis (PCA) are examples of unsupervised learning, which are useful for tasks like clustering and dimensionality reduction. Semi-supervised learning is a mix of supervised and unsupervised learning. It uses a small amount of labeled data along with a large amount of unlabeled data. This method is helpful when labeling data is difficult or expensive, like in healthcare. Finally, reinforcement learning is a method where a model learns by trial and error. It interacts with an environment, gets feedback through rewards or penalties, and uses this feedback to improve its actions over time. While these traditional machine learning approaches have shown significant success [32–35], they require manual feature extraction, as shown in Figure 2. This is where deep learning distinguishes itself.

### Deep learning

Deep learning uses artificial neural networks with multiple layers to automatically extract and learn high-level features directly from raw data, such as images or signals, without requiring human intervention in the feature selection process (Figure 2). This makes deep learning particularly well-suited for tasks involving image recognition, speech processing, and natural language understanding. In the medical field, deep learning has revolutionized diagnostic applications by providing outstanding accuracy in analyzing medical images, such as MRI scans [36, 37], X-rays [38, 39], and retinal images [9, 40].

Deep learning networks, such as CNNs and recurrent neural networks (RNNs), have specific strengths tailored to different types of data. CNNs, for instance, are optimized for image data, employing layers that can automatically detect features such as edges, textures, and shapes in medical images. CNNs are extensively used in tasks like identifying tumors in medical scans [41–43] or detecting retinal abnormalities in ophthalmology [44–46]. On the other hand, RNNs excel at processing sequential data, making them useful for time-series data, such as monitoring disease progression or analyzing electrocardiogram (ECG) signals [47, 48].

Transfer Learning is an essential technique in deep learning that allows models to leverage knowledge gained from one task and apply it to a different but related task. This is particularly useful in medical imaging because large, labeled datasets are often difficult to obtain. A model pre-trained on a large dataset, such as ImageNet [49], learns general image features like edges and shapes. This pre-trained model can then be adapted to a new task, such as detecting tumors in retinal images, by fine-tuning its layers with a smaller dataset specific to the medical application. Transfer learning greatly reduces the amount of labeled data and training time needed while improving model performance in fields like ophthalmology, where data scarcity is a common challenge.

### Evaluation metrics

To assess the performance of both machine learning and deep learning models, several evaluation metrics are commonly used in medical diagnostics. These metrics include accuracy, sensitivity, specificity, F-1 score, and area under the curve (AUC). Explanations of these metrics are given below.

Accuracy: Accuracy measures the proportion of correctly predicted instances (both positive and negative) out of the total instances. It provides an overall measure of how often the model is correct.

$$Accuracy=\frac{TP+TN}{TP+TN+FP+FN}$$

Where TP = True Positives, TN = True Negatives, FP = False Positives, FN = False Negatives.

Sensitivity (Recall or True Positive Rate): Sensitivity measures the model’s ability to correctly identify positive cases (correctly diagnosing a condition when it is present). It indicates how well the model captures actual positives in the dataset.

$$Sensitivity=\frac{TP}{TP+FN}$$

Specificity (True Negative Rate): Specificity measures the model’s ability to correctly identify negative cases (correctly identifying when a condition is not present). Specificity focuses on correctly classifying the negatives and avoiding false positives.

$$Specificity=\frac{TN}{TN+FP}$$

Precision: Precision, also known as Positive Predictive Value, measures the proportion of true positive predictions out of all positive predictions made by the model. It’s important when the cost of false positives is high.

$$Precision=\frac{TP}{TP+FP}$$

F-1 Score: The F-1 score is the harmonic mean of precision and sensitivity. It balances both false positives and false negatives and is particularly useful for imbalanced datasets.

$$F-1\;Score=2\times \frac{Precision\times Sensitivity}{Precision+Sensitivity}$$

AUC: AUC is derived from the receiver operating characteristic (ROC) curve, which plots sensitivity against 1 - specificity. AUC gives an aggregate measure of model performance across all classification thresholds. A higher AUC indicates a better-performing model, with an AUC of 1.0 representing perfect classification.

## Applications of artificial intelligence in choroidal tumor diagnosis

Machine learning and deep learning have recently been studied in the diagnosis of melanocytic choroidal tumors, particularly in differentiating between choroidal nevi and melanomas. These techniques have been applied to clinical datasets [50], pathologic specimens [51], and various imaging modalities, including fundus photography [52–56], OCT [50], and ultrasound imaging [57, 58], to enhance the accuracy and efficiency of diagnosing these tumors. Below, we discuss the use of both machine learning and deep learning models in the context of choroidal tumor diagnosis using ophthalmic imaging.

### Machine learning

Machine learning models have been applied to manually extracted features from a variety of imaging modalities for the diagnosis of melanocytic choroidal tumors. Machine learning models typically rely on the manual extraction of key features, such as tumor thickness, subretinal fluid, orange pigment, the presence of drusen, ultrasonographic hollowness, and morphology of the tumor. These features are recognized as important risk factors for the potential malignant transformation of choroidal nevi into melanoma [18].

For example, Zabor et al. utilized logistic regression models to predict the malignancy of choroidal nevi based on features manually extracted from fundus photographs, OCT, and ultrasound [50]. Their model, which was developed using 123 patients and externally validated on a separate cohort of 240 patients, achieved an AUC of 0.861 in predicting small choroidal melanomas. From their study, their model identified tumor thickness, subretinal fluid, and orange pigmentation as significant risk factors associated with increased odds of malignancy. While these findings align with prior research emphasizing the importance of these features in distinguishing benign nevi from malignant melanomas [17, 19, 59], the performance of machine learning models is inherently limited by the quality and selection of features. The inclusion of external validation in this study is a notable strength, ensuring that the model can generalize to different clinical settings. Additionally, the authors created an online prediction tool to facilitate the real-world application of their machine learning model in clinical practice.

In another study, Jegelevicius et al. employed a decision tree model using features extracted from ultrasound imaging for the differential diagnosis of intraocular tumors [58]. Their results indicated that features such as tumor thickness, base width, and tumor shape were considered important for tumor classification. The decision tree model achieved a diagnostic error rate of 6.7%, demonstrating its potential as a decision-support tool for clinicians. However, as with other machine learning approaches, the reliance on manually extracted features and the inherent limitation of focusing only on predefined criteria restricts its scalability and generalizability.

One of the primary limitations of traditional machine learning in this context is its dependence on human-driven feature extraction. While features like tumor size or subretinal fluid are easily identifiable and quantifiable, these models struggle to detect more complex patterns that may be key to differentiating between benign and malignant lesions. As a result, machine learning models can be less effective for tumor diagnosis compared to deep learning models, which can automatically learn and extract intricate hierarchical features from raw imaging data.

### Deep learning

Deep learning has made substantial progress in diagnosing choroidal tumors, primarily through the use of CNNs. The power of CNNs lies in their ability to automatically learn features from raw images without the need for manual feature extraction, making them ideal for analyzing medical images, particularly in diagnosing ocular conditions such as uveal melanoma.

Shakeri et al. demonstrated the efficacy of CNNs in the detection of uveal melanoma using fundus images [60]. They employed pre-trained models like DenseNet121, DenseNet169, Inception-V3, and Xception, using transfer learning to fine-tune the models on the fundus images. Their best-performing model, DenseNet169, achieved an accuracy of 89%.

Similarly, Ganguly et al. achieved similar results using a custom CNN model trained on fundus images. Their model achieved an accuracy of 92%, an F1-score of 0.93, and a precision of 0.97 [54]. However, the relatively small dataset of 170 images used in the study presents concerns about overfitting, where the model may perform well on training data but struggle to generalize to new, unseen data.

Additionally, Hoffmann et al. utilized ResNet50 to distinguish choroidal melanoma from choroidal nevi using fundus images [55]. They reported an accuracy of 90.9% and F1 score of 0.91, and an AUC of 0.99, showcasing the high potential of deep learning models in automating tumor differentiation tasks. Furthermore, they showed that deep learning models can be used to estimate the likelihood of malignancy in melanocytic choroidal tumors.

In another study, Dadzie et al. focused on enhancing deep learning performance through color fusion strategies [56]. By employing the DenseNet121 architecture on ultra-widefield retinal images, they examined how combining color channels (red, green, and blue) affect tumor classification accuracy. Their results showed that the intermediate fusion provided the best classification accuracy, outperforming early and late fusion strategies with an accuracy of 92.2%, an F1 score of 0.88, and an AUC of 0.98. This study highlighted the importance of leveraging different color channels in fundus images to maximize the potential of deep learning models in tumor classification. The integration of multiple color channels enables these models to exploit more detailed color and texture information, further refining their classification capabilities. While color fusion is a novel approach, the study did not address the computational cost of using different fusion strategies which is an important consideration for clinical deployment.

In a recent study by Sabazade et al, they tackled a critical challenge in deep learning for choroidal melanoma diagnosis by ensuring model generalizability through a multicenter approach [52]. They used a custom U-Net architecture that achieved an average F1- score of 0.77 and an average AUC of 0.89 for the test dataset. On external validation dataset, the model achieved an F1-score of 0.71 and an AUC of 0.88. Notably, accuracy was not reported in this study. By incorporating datasets from various centers and imaging devices, they reduced the risk of bias often seen in single-center studies and improved the model’s robustness. The inclusion of external validation was a crucial step toward real-world applicability, as it demonstrated the model’s ability to generalize beyond the original training dataset. However, despite addressing generalizability, the study relied on relatively smaller datasets, which can still pose a risk of overfitting.

Addressing the issue of small datasets, another recent study by Jackson et. al employed over 25,000 ultra-widefield retinal images for deep learning classification of choroidal melanoma and nevus [53]. Using a transfer learning approach, they employed the RETFound, a foundation self-supervised deep learning model [61]. Their model achieved an accuracy of 83%, an F1 score of 0.84, and an AUC of 0.90. While the study demonstrates a promising solution to data scarcity, the lack of external validation limits its immediate clinical applicability, as model performance across different populations and imaging devices remains uncertain.

The studies reported above collectively demonstrate the powerful role that deep learning models play in the detection and classification of choroidal melanoma using fundus images. However, several limitations remain across the studies, such as the use of small datasets, the lack of external validation, and the reliance on a single imaging modality. However, recent studies have made progress in addressing these issues [52, 53], but further research is needed to explore the integration of multimodal imaging, which could offer a more comprehensive diagnostic approach. Table 1 offers a detailed comparison of the methodologies and results across these studies, providing a clear overview of how different architectures and approaches enhance tumor differentiation. The models presented in Table 1 represent the best-performing models reported in each study.

**TABLE 1:** Comparison between studies that used deep learning for the classification of Choroidal Nevus and Melanoma.

Study	Model used	Imaging modality	Number of Images			Performance metrics					
			Control	Melanoma	Nevus	Accuracy	Sensitivity	Specificity	Precision	F1-Score	AUC
Ganguly et al. [54]	Custom model	Standard images	NA	110	60	0.92	0.90	0.95	0.97	0.93	NA
Hoffman et al. [55]	ResNet50	UWF and standard images	NA	422	340	0.91	0.90	0.91	0.91	0.91	0.99
Dadzie et al. [56]	DenseNet121	UWF images	360	157	281	0.92	0.81	0.98	0.96	0.88	0.95
Sabazade et al. [52]	Custom model	UWF and standard images	NA	219	583	NA	1.00	0.74	NA	0.77	0.89
Jackson et al. [53]	RETFound	UWF images	1,192	18,510	8,671	0.83	0.79	0.87	0.89	0.84	0.90

UWF, Ultra-widefield; NA, not available.

While fundus photography has been extensively used in deep learning applications for classifying melanocytic choroidal tumors, other imaging modalities, such as OCT and ultrasound, have not been widely employed for this purpose. This gap presents an opportunity for future research to explore the application of deep learning to these imaging modalities, which could enhance early detection and diagnosis of uveal melanoma by integrating structural information from OCT and ultrasound with surface-level data from fundus photography.

## Challenges and limitations

The application of artificial intelligence in diagnosing choroidal melanoma has shown considerable promise, demonstrating the potential for enhanced diagnostic accuracy and efficiency. However, translating these advancements from research settings into routine clinical practice is accompanied by significant challenges and limitations. As machine learning and deep learning models evolve, understanding and addressing the obstacles that limit their clinical integration becomes critical, particularly for supporting downstream diagnosis and aiding non-specialists. These challenges encompass a range of issues, from the quality and availability of data to the interpretability of model outputs and the adaptability of machine learning systems within clinical workflows.

Effective implementation of artificial intelligence tools in healthcare requires not only technological advancements but also a nuanced understanding of clinical requirements and patient variability. Machine learning and deep learning models must be rigorously validated, reliable across diverse patient populations and imaging techniques, and transparent in their decision-making processes. Given the known differences in the incidence of choroidal nevi and choroidal melanoma across racial and ethnic groups, datasets used to train these models are often disproportionately composed of images from populations with higher disease prevalence, primarily White individuals. This imbalance can introduce bias, potentially limiting the model’s applicability to underrepresented populations. Addressing these aspects is essential to achieving widespread clinical adoption and ensuring that artificial intelligence technologies fulfill their potential to improve patient outcomes. The following sections outline key challenges and limitations, providing insights into areas that require further research and refinement to support the successful deployment of machine learning in ocular oncology. Table 2 summarizes the main challenges and limitations in applying machine learning to uveal melanoma diagnosis, along with potential solutions to address each issue.

**TABLE 2:** Challenges and potential solutions for integrating machine learning in uveal melanoma diagnosis.

Challenge	Description	Implication	Potential solutions
Data Availability	Uveal melanoma is rare, making it difficult to acquire large datasets for training robust models	Limited dataset size restricts model accuracy, leads to overfitting, and reduces model generalizability	Data-sharing collaborations; multimodal image training to optimize models using available data; synthetic data generation
Dataset Diversity	Many datasets are from single institutions or regions, limiting diversity in patient demographics and imaging conditions	Models trained on specific populations may not perform well across different demographics, leading to bias in predictions	Multicenter collaborations; access to data from clinical trials; external validation to ensure generalizability
Ground Truth Labeling	While standardized systems like MOLES and histopathological analysis exist, their adoption varies globally, posing challenges for consistent ground truth labeling	Lack of standardization in labeling can affect model accuracy and interpretability. Model design may not align with the intended clinical use	Broader adoption of standardized systems and expert consensus on defining ground truths can improve consistency in labeling across studies. Additionally, intraocular biopsy results, when available, can provide more definitive labels
Model Interpretability	Deep learning models often have opaque decision-making processes, making it difficult to understand exactly which features drive predictions. While techniques like CAMs, including Grad-CAM highlight regions used by the model, they do not reveal the specific features influencing the decision	Lack of transparency limits clinician trust and makes clinical integration difficult	Techniques such as CAMs and SHAP provide visual and feature-level explanations. Further improvement of these methods can enhance model transparency, build clinician trust, and support clinical integration
Clinical Integration	Machine learning tools often function as stand-alone systems, lacking integration with EHRs and imaging platforms	Increase workload for healthcare providers, as they may need to navigate multiple platforms to incorporate machine learning insights with traditional methods	Development of interoperable systems that integrate directly with EHRs and imaging systems for seamless workflow
Ethical Considerations	Use of machine learning algorithms for life-altering decisions like diagnosis and treatment raises ethical concerns	High-stakes medical decisions by machine learning must meet rigorous standards, as errors can seriously impact patient health	Rigorous validation of machine learning outputs with clinical ground truths and use as a decision support tool rather than for sole decision-making

GAN, generative adversarial network; CAM, class activation mapping; Grad-CAM, Gradient-weighted Class Activation Mapping; SHAP, Shapely Additive exPlanations; EHR, electronic health records.

### Data availability and quality

One of the most significant challenges in developing effective machine learning and deep learning models for the diagnosis of melanocytic choroidal tumors is the availability and quality of data. Uveal melanoma is a rare condition, making it difficult to acquire large, diverse datasets that are essential for training robust machine learning models. The rarity of the condition results in relatively small datasets, which can lead to overfitting in models. This issue is especially problematic in deep learning, where large-scale datasets are crucial for capturing complex patterns in medical images and achieving high performance. A recent study by Jackson et al. has begun addressing this issue by utilizing large datasets with over 25,000 images [53].

Another issue related to data limitations is the lack of diversity in the available datasets. Often times, datasets are collected from a single institution or geographic region, limiting the model’s exposure to different patient demographics, imaging conditions, and equipment variations. A model trained on data from a specific population may not perform well when applied to a different demographic, leading to bias in predictions and unequal outcomes across patient populations. Potential solutions to these limitations include data-sharing collaborations between institutions and leveraging images being collected for the increasing number of prospective multi-center clinical trials being conducted for uveal melanoma. Another promising avenue for addressing the issue of data scarcity is the use of synthetic data. Generative adversarial networks (GANs) and diffusion models have emerged as tools for generating synthetic medical images that mimic real-world data [62–65]. These synthetic datasets can be used to augment existing data, allowing deep learning models to train on larger and more diverse datasets. However, synthetic data may raise concerns about trust, as models trained on artificially generated images could be perceived as less reliable for real-world clinical applications. Therefore, proper validation on real-world data is essential to build clinician and patient confidence in these models.

### Ground truth labeling and standardization

Another challenge regarding the dataset involves ground truth labeling. There are no universally accepted criteria for the definition of choroidal nevi and melanoma, which is why some cases are called indeterminate until the tumor is observed to be stable in size, grow during the observation period, or occasionally biopsied. Most often, the diagnosis of choroidal melanoma is clinical, but more careful definitions should be chosen for the indeterminate cases. Choroidal nevi versus melanomas can be defined by its clinical appearance, its pathological appearance (when tissue is available), or its metastatic potential and disease-specific mortality when long-term follow-up is available. The labels for ground truths in machine learning and deep learning studies should be chosen carefully to reflect the dataset available for that study. Alternative labels may include the tumor size, clinical characteristics, genetic profile, tumor growth rate over time, or the management decision chosen by the clinician. The type of machine learning project and clinical goal of the study should determine the ground truth labels being chosen, and care should be taken to prevent conclusions about an artificial intelligence model that exceeds the scope of the available data. Potential solutions to this challenge include expert committees to standardize the definitions and labels being used for machine learning studies in ocular oncology. Additionally, the MOLES scoring system, which is widely used in clinical practice for risk stratification of choroidal nevi, could serve as a standardized framework for developing ground truth labels in artificial intelligence studies. Labels agnostic of management decisions, such as calling the same lesion indeterminate when it would be observed versus a small melanoma when it would be treated, can also improve the generalizability of the study when opinions on management differ.

### Model interpretability

Another significant challenge in the adoption of machine learning and deep learning models in the diagnosis of melanocytic choroidal tumors is the lack of interpretability. While deep learning models have shown exceptional performance in classifying uveal melanoma and other ocular conditions, their decision-making process remains largely opaque. This is often referred to as the black box nature of deep learning. This lack of transparency often makes these models difficult to trust and integrate into clinical practice, as clinicians typically need to understand the reasoning behind a model’s prediction to confidently use it for patient care.

**FIGURE 3:**
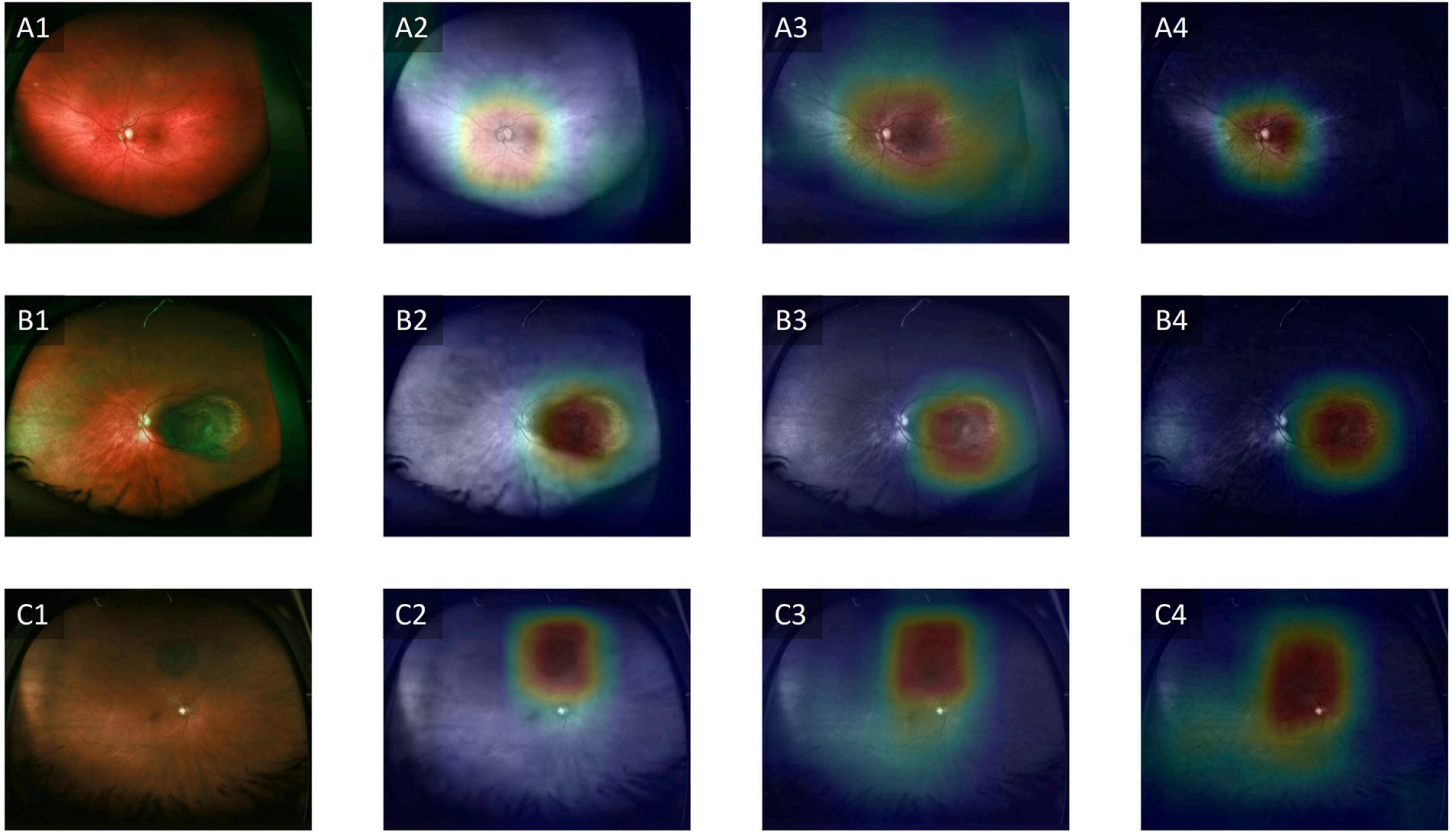
Grad-CAM showing regions relevant for classification. **(A1–A4)** Control, **(B1–B4)** Choroidal melanoma, **(C1–C4)** Choroidal nevus. Column 1 represents the original images, column 2 represents the red channels, column 3 represents the green channels and column 4 represents the blue channels. Adapted from [67].

**FIGURE 4:**
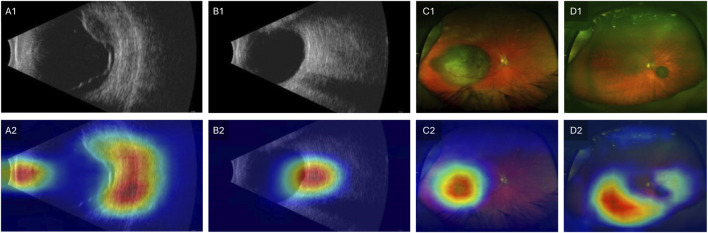
Grad-CAM showing regions relevant for classification. Row 1 shows original images and Row 2 shows the Grad-CAM heatmaps overlaid on the original images. **(A1–A2)** Ultrasound image of choroidal melanoma. **(B1–B2)** Ultrasound image of choroidal nevus. **(C1–C2)** Ultra-widefield retinal image of choroidal melanoma. **(D1–D2)** Ultra-widefield retinal image of choroidal nevus. Modified with permission from [57].

To address the black-box issue in deep learning, researchers have developed several techniques to enhance the interpretability of these models in medical imaging. These methods provide insights into how the model makes predictions and identifies which parts of the image are most influential in the decision-making process. A commonly used method is Gradient-weighted Class Activation Mapping (Grad-CAM), which generates heatmaps overlaid on the original image to show which regions contributed the most to the model’s classification [66]. Grad-CAM works by leveraging the gradients of a target class flowing into the final convolutional layer of a neural network, which produces a localization map highlighting the important regions in the image [66]. Clinicians can cross-reference the model’s focus areas with their own knowledge of disease markers, ensuring that the model is not relying on irrelevant features. Another advantage of improved interpretability is the potential for clinicians to learn new insights into image evaluation. Experts can potentially learn new clinical or imaging features from machine learning models, to improve our understanding of how best to interpret imaging to diagnose melanocytic tumors. Figure 3 illustrates an example of Grad-CAM heatmap overlaid on different color channels of ultra-widefield retinal images to show regions considered most relevant for classification of choroidal nevus and melanoma. Figure 4 also shows the areas in ultrasound images that are considered crucial for deep learning models to make a classification decision.

### Integration into clinical workflow

Another key challenge in the adoption of deep learning and machine learning models for the diagnosis of melanocytic choroidal is their integration into existing clinical workflows. While these models have demonstrated impressive accuracy in research settings, translating them into real-world clinical practice remains difficult. Many machine learning tools function as stand-alone systems that do not seamlessly integrate with existing electronic health records (EHRs) or imaging systems, causing potential disruptions in established processes. This lack of interoperability can increase the workload for healthcare providers, who may need to navigate between multiple platforms to interpret machine learning results alongside traditional diagnostic methods. However, tools like the online calculator developed by Zabor et al. demonstrate how these models could be applied in practice [50]. Although the current version requires manual input, future versions could be integrated directly into clinical workflows, automatically extracting data from existing databases and providing instant results.

### Ethical considerations

Another challenge in the clinical application of artificial intelligence in the diagnosis of uveal melanoma is the degree to which physicians will rely on machine learning algorithms. In cases of indeterminate choroidal tumors, diagnosis using histopathologic examination has been reported to be upwards of 90% accuracy [68, 69]. This level of accuracy will likely be surpassed by machine learning algorithms using multimodal data, depending on the quality of labels and choice of ground truth for the diagnosis of uveal melanoma. However, the ethical implications of using a computer algorithm to make vision and life-threatening decisions raise the standard for the required accuracy and quality of validation for this clinical use. In the near term, machine learning and deep learning algorithms may be used as clinical tools, such as for screening and triaging by optometrists and comprehensive ophthalmologists, or aid in management decisions along with clinical data, imaging, genetics, and biopsy results when deemed necessary. These tools are likely to be more beneficial in non-specialist community centers, where diagnostic accuracy may be lower, rather than in specialist ocular oncology centers, where experienced clinicians already achieve high diagnostic accuracy. The latter use as a tool for ocular oncologists will become more useful as long-term follow-up data is available to validate algorithm outputs prospectively with clinically meaningful ground truths, including tumor growth rate, histopathologic findings, metastatic risk, and disease-specific mortality.

## Discussion

The application of artificial intelligence to diagnose melanocytic choroidal tumors represents a promising advancement in ocular oncology. Machine learning methods rely on manual examination of imaging features, which introduces variability and often limits diagnostic accuracy. Deep learning models, especially CNNs, address some of these limitations by automating feature extraction and analysis, improving diagnostic reliability and potentially enhancing patient outcomes.

Studies have predominantly utilized fundus photography as the primary imaging modality for developing and validating machine learning models. Pre-trained CNN architectures and custom-built CNNs have already achieved accuracy metrics surpassing 90%, demonstrating the effectiveness of these models in capturing key image features. Despite these promising findings, models trained exclusively on fundus photography have inherent limitations. Fundus images provide a surface-level view, which may not capture critical information such as tumor depth and structural details. In contrast, OCT and ultrasound are capable of capturing deeper, three-dimensional characteristics of tumors, offering a more comprehensive understanding of tumor morphology.

By combining these different modalities, deep learning models can access a richer and more diverse set of features, enabling more accurate diagnosis and improving the ability to detect subtle signs of malignancy. Multimodal imaging has shown considerable success in ophthalmology, particularly in the diagnosis and management of conditions like diabetic retinopathy [70, 71], age-related macular degeneration (AMD) [72, 73], and glaucoma [74, 75]. However, despite the demonstrated success of multimodal imaging in diagnosing retinal diseases, its application to choroidal tumors has been limited. The use of multi-modal imaging for choroidal tumors has the potential to significantly enhance diagnostic accuracy by merging surface-level information from fundus photography with cross-sectional structural details provided by OCT and internal characteristics captured through ultrasound. Each modality offers unique advantages: fundus photography captures surface features like lesion color and margins, OCT provides detailed tumor thickness and subretinal fluid information, and ultrasound allows for the assessment of thicker tumor depth and internal echogenicity.

Such multimodal approaches hold wide-ranging clinical applications, including screening, referral triaging, risk assessment, detailed tumor characterization for clinical trials, surveillance, planning of radiation therapy, and monitoring for local recurrence after treatment. Multimodal imaging thus represents a promising frontier for research and innovation in ocular oncology, supporting the continued refinement of machine learning and deep learning tools in diagnosing and managing uveal melanoma.

Moreover, issues related to data availability and quality remain a major hurdle. Uveal melanoma is a rare condition, and the lack of large, diverse datasets increases the risk of model overfitting and reduces generalizability. Recent efforts to mitigate these issues include multicenter collaborations and external validation, which have demonstrated improved model robustness across different populations and imaging conditions [52]. Another significant challenge lies in clinical integration. While machine learning and deep learning models have achieved impressive results in research environments, real-world deployment requires seamless integration with electronic health records and imaging systems. This integration is crucial for reducing the workload on clinicians and ensuring that artificial-intelligence-generated insights can be readily incorporated into routine clinical workflows. Tools such as online prediction calculators have shown promise in this regard, but further work is needed to automate data input and output processes [50].

## Conclusion

Artificial intelligence has the potential to improve the diagnostic approach to melanocytic choroidal tumors, offering significant improvements in accuracy and efficiency. However, challenges related to data availability, ground truth labeling, model interpretability, and clinical integration must be addressed to fully realize the potential of these tools in everyday clinical practice. Future directions such as the use of collaborative networks of institutions, multimodal imaging integration, and improved interpretability methods are key to overcoming current limitations. As these technologies continue to evolve, they are expected to play an increasingly important role in uveal melanoma diagnosis and treatment, leading to earlier detection, more accurate diagnoses, and ultimately better patient outcomes. Possible clinical applications encompass screening, referral triage, diagnosis, risk assessment, tumor localization and morphological analysis for clinical trials, active surveillance, radiation planning, and monitoring for post-treatment recurrence. Several critical areas require further exploration and development to fully realize the potential of these technologies. With continued refinement and targeted solutions to current limitations, machine learning and deep learning diagnostic tools could become integral to ophthalmic practice, empowering clinicians to make more accurate and informed decisions.
